# Hemostatic palliative radiotherapy for gastric cancer: A literature review

**DOI:** 10.1016/j.tipsro.2024.100266

**Published:** 2024-07-31

**Authors:** Osamu Tanaka

**Affiliations:** Asahi University Hospital, Department of Radiation Oncology, Gifu, 500-8523, Japan

**Keywords:** Radiotherapy, Gastric cancer, Hemostasis, Palliation, Reirradiation

## Abstract

**Background:**

Gastric cancer has a high prevalence in Asia and may only be diagnosed in advanced stages. Therefore, patients with gastric cancer may experience fatal symptoms, such as bleeding or stenosis at the time of consultation. In this review, we aimed to describe the effectiveness and toxicity of hemostatic radiotherapy (RT).

**Methods:**

A total of 17 retrospective and 3 prospective studies were analyzed. The prescription dose, biologically effective dose, equivalent dose in 2 Gy fractions, response rate, survival prognosis, and toxicities were also reported.

**Results:**

Using 20 studies, the following observations were made the hemostatic effect was ∼ 80 %, the mean survival time after irradiation was about 3 months, and prescribed doses of 30 Gy/10 fractions and 20 Gy/5 fractions were considered suitable.

**Conclusion:**

In this review, studies on hemostatic irradiation have been summarized, and the most optimal treatment method has been proposed. 30 Gy/10 fractions and 20 Gy/5 fractions were ideal. However, because palliative RT is preferably completed within a short period of time, a randomized trial is needed to determine whether the 8 Gy/single fraction treatment is equivalent to fractionated RT. Therefore, more prospective studies are warranted to establish a standard of care for palliative RT in gastric cancer.

## Highlights

• Palliative radiotherapy is rarely used for the treatment of gastric cancer.
• Standard treatment for inoperable advanced gastric cancer is not established yet.
• Low doses of approximately BED10 of 30 Gy (EQD2 of 24 Gy) exert hemostatic effects.
• Three prospective trials have been reported and more prospective trials are needed.

## Introduction

Gastric cancer is commonly diagnosed at advanced stages and often accompanied by bleeding. Its management includes endoscopic therapy, surgery, chemotherapy, and radiotherapy. However, a consensus on treatment strategies has not been established. Conversely, radiation therapy is noninvasive and its utility has been reported in previous retrospective studies.

Palliative radiotherapy (RT) is rarely used for the treatment of gastric cancer; however, pre- or postoperative RTs are commonly administered in Europe and the United States [1], [2], [3], [4], [5], as it is noninvasive and a highly effective radical treatment.

Generally, palliative RT dose is lower than the radical RT dose, with lower chances of side effects. Thus, the purpose of RT should be properly understood. Bleeding in stomach cancer cannot be completely stopped using palliative RT. The efficacy of hemostatic RT against bleeding from the head and neck, bronchi, rectum, bladder, and uterus has already been reported [6].

The standard treatment for inoperable advanced gastric cancer has not yet been established because the treatment method is selected after assessing the irradiation method and results of retrospective studies. Moreover, only a few prospective studies are currently available. This review describes the current evidence available on palliative hemostasis irradiation for gastric cancer and the direction of future approaches for palliative irradiation in cases of gastric cancer [7], [8], [9], [10], [11], [12], [13], [14], [15], [16], [17], [18].

## Material and methods

**Fig. 1:**
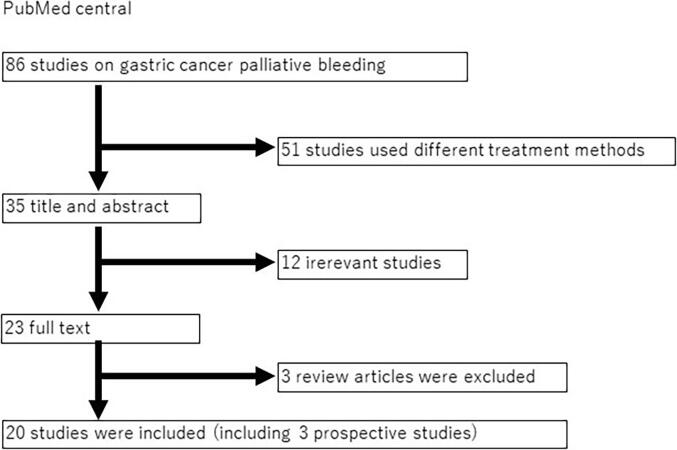
Details of the search strategy We found 20 non-comparative observational studies including patients who received hemostatic radiotherapy for the treatment of local bleeding.

A tree of selections and criteria is shown in Fig. 1**.**

Literature search was carried out on the PubMed database, selecting data between 2007 to present using the following relevant keywords: “Gastric Cancer,” “Bleeding,” “Radiotherapy,” and “Palliation” (Fig. 1). Surveillance performed within Japan was excluded from this review [17]. Articles written in languages other than English and case reports were also excluded. Previously, review articles have been reported, however, no prospective studies are included (prospective studies have been reported since 2019). Viani et al. reviewed 11 studies, including a prospective by Tey et al., but not by Tanaka (2020) and Saito (2021). A 2022 review article by Kawabata only examined 10 cases published since 2017. At present, no research has reviewed all reports from 2007 to 2022. Therefore, this review of retro- and prospective observational studies aimed to prepare material for future randomized trials.

### Setting the end-point for gastric cancer hemostasis

As with all types of palliative medicine, determining the end-point of the treatment is essential, including symptom relief, increased time spent at home owing to discharge, and survival duration [8], [9], [10], [11], [12]. Because several previous retrospective studies were available for review, medical record changes based on the quality of life (QoL) and survival were evaluated. However, the results often do not reflect the patients’ real prognosis because several limitations were associated with these retrospective studies, making it difficult to set an end-point for diseases with a short prognosis. We therefore describe the evaluation outcomes separately in each report.

## Results

The response shows the rate at which the hemostatic effect was obtained (Table 1**)**. Only prospective studies by Saito et al. estimated QoL changes, which were not considered in this literature review. Evaluation of strictures was not covered in this review because of the inconsistent assessment criteria; however, several reports have demonstrated successful stenosis release.

**Table 1:** Characteristics and results of hemostatic radiotherapy for gastric cancer.

Authors	Cases	BED10 (Gy)	EQD2(10)	Response rate (evaluation definition)	MST (month)	RT planning	Reirradiation	Toxicity > Grade 3	Reference no.
Tey J (2007)#	24/33	39	32.5	54 % (13/24) relief from bleeding, obstruction, pain	4.8	3DRT	None	2 cases	[7]
Kim (2008)#	20/37	43.5	36.4	70 % (14/20) relief from bleeding, obstruction, pain	5.2	3DRT	None	7 cases	[27]
Hashimoto (2009)	19	50	41.6	92 % (11/12) overall survival	3.4	3DRT	None	4 cases	[14]
Lee JA (2009)	23	39	32.5	91 % (21/23) relief from bleeding, overall survival	N/A	2DRT	None	None	[12]
Asakura (2011)	30	39	32.5	73 % (22/30) Hemoglobin, transfusion	3.6	3DRT	None	3 cases	[15]
Chaw (2014)	52	14.4	12	50 % (22/44) Hemoglobin, transfusion	5.3	3DRT	3 cases	N/A	[13]
Tey J (2014)	115	39	32.5	80 % (83/103) relief from bleeding, obstruction, pain	2.8	3DRT	None	3 cases	[9]
Kondoh (2015)	15	39	32.5	73 % (11/15) Hemoglobin, melena	2.1	3DRT	None	5 cases	[16]
Kawabata (2017)	18	7.2	6	55 % (10/18) hemoglobin	N/A	3DRT	None	None	[10]
Lee YH (2017)	42	48 or 27.3	57.6 or 22.7	69 % (29/42) relief from bleeding	3.1	3DRT	None	None	[8]
Hiramoto (2018)	23	50.4	42	89 % (16/18) relief from bleeding	3.9	3DRT	None	None	[28]
Lee J (2021)	57	37.5	31.2	75 % (43/57) Hemoglobin, transfusion	3.4	3DRT	None	None	[29]
Yu J (2021)	61	39	32.5	88 % (54/61) Hemoglobin, rebleeding	4.8	3DRT	None	1 case	[30]
Mitsuhashi (2021)	28	39 or 48	32.5 or 57.6	N/A Hemoglobin, transfusion, overall survival	¶	3DRT	None	1 case Grade 5	[31]
Takeda (2022)	120	39	32.5	59 % (71/120) overall survival	3.7	3DRT	3 cases	6 cases	[32]
Sugita (2022)	33	39	32.5	73 % (24/33) Hemoglobin, transfusion, melena	3.7	3DRT	None	None	[33]
Kawabata (2022)	20	39	32.5	95 % (19/20) Hemoglobin, transfusion	12	3DRT	3 cases	1 cases	[25]
Tey J* (2019)	50	46.8	39	80 % (40/50) fatigue, dysphagia, pain	2.8	3DRT	None	2 cases	[19]
Tanaka* (2020)	31	28	23.3	80 % (25/31) overall survival	3	3DRT	6 cases	None	[21]
Saito* (2022)	55	28	23.3	69 % (38/55) relief from bleeding, quality of life	2.3	3DRT	None	1 case	[20]

*Prospective studies; # Irradiated for not only patients with bleeding but also with obstruction and pain. Patients with bleeding were 24/33 and 20/37. ¶ Overall survival rates in all patients 12%.

BED: biological effective doseα/β = 10; EQD2: equivalent dose in 2 Gy fractions a/b = 10; MST: median survival time; RT: radiotherapy; 3D: three dimensional.

### Dose calculations

The linear-quadratic model is commonly used in radiotherapy units and allows for easy analysis of equivalent dose for different fractionations. Equivalent dose in 2 Gy fractions (EQD2) is the dose obtained using a 2 Gy fraction dose, which is biologically equivalent to a total dose D (dose) given with a fraction dose of Gy. Moreover, the EQD2 values may be added in separate parts of the treatment plan and may be adapted in fraction doses other than 2 Gy. In our 20 reviews, biologically effective dose 10 (BED10) was the most common at 30–40 Gy, and EQD2 (10) was mostly at 30 Gy.

Lee et al. reported that a high response rate was achieved when BED regimens (α/β ratio = 10) had exceeded 36 Gy [8], whereas some published reports like the study by Tey demonstrate good therapeutic effect at 39 Gy [7]. In Tey’s study, RT regimens’ dosages ranged from an 8 Gy single fraction to 40  Gy/16. The majority of patients (46/115) received 30  Gy/10 fractions delivered at 3  Gy/fraction, five fractions per week. Their response rates to bleeding were 80.6 %, and no difference was observed between low (≤39 Gy) and high (>39 Gy) doses in BED regimens (α/β ratio = 10).

The search results of the prospective studies are shown below:

1) Tey et al. 36 Gy/12 fractions fixed prospective study [19].
2) Saito et al. from the Japan Radiation Oncology Study Group (JROSG 17–3) [20].
3) Tanaka et al. 20 Gy/5f fraction fixed prospective study. UMIN-CTR registration number UMIN000026362 [21].

Tey et al. analyzed the QoL of patients mainly treated with fixed irradiation of 36 Gy/12 [19]. The primary outcomes were symptom response rates. Secondary outcomes covered overall survival (OS). Fifty patients were accrued in their study, and median survival duration was 85 days. Their primary outcomes were the alleviation of fatigue, nausea, and pain subscales of the EORTC QLQ‐C30. Those results were seen in 50 %, 28 %, and 44 % of patients at the end of RT and in 63 %, 31 %, and 50 % of them 1 month after the RT. Their study shows that palliative gastric RT is effective and well-tolerated, while it allowed alleviation of fatigue, dysphagia, and pain at the end of the RT and 1 month after the completion of the RT in a significant proportion of patients.

Saito et al. also emphasized on the QoL without fixing the prescription dose (8 Gy single dose, 20 Gy/5 fractions, and 30 Gy/10 fractions) [20]. Their treatment response rates were 47 %, 53 %, and 49 % at 2, 4, and 8 weeks of follow-up, respectively. However, neither response nor BED (α/β = 10) predicted OS. Univariate cox model showed that BED was not significantly associated with rebleeding. This result is similar to those of previous retrospective reports, indicating that BED was not related to bleeding control. However, they found that the per-protocol response rate increased to 90 % during the 8-week follow-up. They concluded that the above protocol is useful, although the appropriate BED was unknown.

Conversely, in Tanaka et al.’s study, repeated irradiation was examined with an emphasis on bleeding, using a fixed prescription of 20 Gy/5 fx for the entire stomach [21]. The response rate of initial the RT was 80 % (25/31), and 6 of the 25 patients underwent repeated irradiation; all 6 were responders (100 %) for the prescribed 15 Gy/5 fx for partial irradiation of the stomach. The median OS was significantly different among the entire cohort and between one-time and repeated irradiation groups (91, 76, and 112 days, respectively).

Thus, even in a prospective study, successful treatment depends on the end-point used in the trial because the interpretation of results becomes more complicated based on the QoL, survival time in palliative irradiation, and recurrence-free survival time.

## Discussion

The purposes of palliative treatment widely varied in the assessed studies and are as follows: 1) QoL improvement, 2) extension of survival time, and 3) extension of the asymptomatic period. However, determining the aspects of palliative treatment that are beneficial to a patient is currently impossible [24], [25]. Hemostatic irradiation is extremely beneficial to patients and should be investigated in detail whenever possible. However, there were some limitations in investigating the effect of hemostatic RT, because the end-points determined in each report were different, e.g.:

1) The parameters of a successful treatment, such as hemoglobin (Hb) levels are preset (several studies use an Hb level of 8.0 g/dl), and hemostatic treatment, for endoscopic treatment and surgery, is generally provided when Hb levels are < 8.0 g/dl. Hemostatic effect is defined as treatment success if Hb remains > 8 g/dl 1 month post-irradiation. However, the definition of hemostatic effects varies depending on the paper, and most studies set the Hb value to ∼ 8 g/dl. RT is also indicated for hemostatic irradiation when Hb levels consistently have values < 8.0 g/dl. However, if Hb levels return to ≤ 8 g/dl post-transfusion, the bleeding did not spontaneously stop. The evaluation method covers an easy-to-understand parameter (Hb level of 8 g/dl) that assesses the extent of the Hb value increase before irradiation or whether the Hb value at pretreatment can be maintained. In retrospective examinations, end-point has not been set; thus, an accurate assessment is considered difficult. Moreover, the patient can be discharged after the bleeding has been stopped. Furthermore, gastrointestinal tract stenosis can be ameliorated using RT.
2) The survival time cannot be compared between the treated and untreated groups.
3) QoL can be determined through the parameters that quantitatively scores a patient’s condition, which is difficult to conduct retrospectively using QoL improvement score [22]. Moreover, accurately evaluating QoL is complicated as the data of retrospective studies are inferred from medical records and scored accordingly. Because of the difficulties associated with these studies, prospective studies should be also included in the future review. As of 2022, three prospective studies have been conducted, and we present a summary of previous reports in the literature shown below.

Conversely, several positive outcomes of treatment have been reported, such as those by Song et al. [23], who evaluated the clinical outcomes of endoscopic hemostasis for bleeding in patients with unresectable gastric cancer. Successful initial hemostasis was attained in 83 % of the patients, and rebleeding occurred in 28.3 % of the patients within 30 days [7], [8], [14], [21], [22]. Hemostasis is achieved using a more noninvasive method than endoscopic technique or surgical bypass surgery.

Based on the reports on different treatment methods, the evidence to date indicates that hemostatic RT is sufficiently effective. The “Gastric cancer: ESMO Clinical Practice Guideline for diagnosis, treatment and follow-up” [26] only mentions preoperative RT; however, palliative RT has been proven to be effective for the treatment of gastric cancer with bleeding. Hemostatic RT should be considered in the guidelines. Symptoms, disease-free survival, OS time, and QoL may be important considerations for patients in palliative care. Although only a few reviews encompassing prospective studies have been conducted, further evidence on palliative irradiation may serve as a guide to favorable outcomes. However, conducting a comparative study on treated for bleeding and untreated patients is questionable. Reviews of previous retrospective studies demonstrated that the hemostatic effect has been found effective, and the assignment to no-treatment may be at a patient’s disadvantage. The purpose of palliative irradiation is not to treat cancer but to ameliorate symptoms. Furthermore, palliative medicine should be as minimally invasive as possible, and the treatment is expected to be completed in a short time.

Tey et al. also reported no difference between doses higher and lower than 39 Gy and concluded that high doses are non-essential. Low dose also useful for patients to be treated in a short time, such as 20 Gy/5 fx [21], because patients who receive palliative irradiation often have a short survival time. Additionally, when endoscopic hemostasis cannot be achieved, surgery or radiotherapy is an option. However, radiotherapy is often more suitable than surgery for palliative medicine with a short survival prognosis. Therefore, reducing the number of hospital visits as much as possible would be useful for patients. Because a single irradiation dose of 8 Gy can also provide the desired effects, such methods are also effective for patients who have difficulty in irradiation positioning setting. For example, if a patient resides far from the hospital, they may face difficulty in shifting to a RT department because of their condition [20]. As a result of these retro- and prospective studies, the established irradiation dose was controversial. Although some studies concluded that increased doses proved effective, others did not. Currently, the correlation between the median BED10 and the hemostasis rate is unclear, and if there is no correlation, a dose with high short-term efficacy should be selected. Furthermore, considering the organ at risk (OAR) dose around the stomach, if repeated irradiation becomes necessary, it would be better to lower the OARS dose for the first irradiation dose.

If the irradiation field can be observed using cone beam computed tomography, it is acceptable to reduce the planning target volume margin as the former can be narrowed and the dose administered to the liver and kidney can be reduced. Moreover, if peristalsis can be suppressed by butyl scopolamine, the accuracy will be even higher.

GRID radiotherapy is used for palliative radiotherapy to treat symptoms associated with cancer and is a radiation therapy that is sometimes administered in conjunction with conventional/three-dimensional conformal radiation therapy. This method is useful when the cancer has metastasized, and the target is large.

## Conclusions

Based on the evidence reported to date, it is evident that even low doses of approximately BED10 of 30 Gy exert hemostatic effects. However, only three prospective trials have been reported. Thus, in the future, more prospective studies should be performed to establish a standard treatment for palliative irradiation for gastric cancer. RT is very important in palliative care. Therefore, it is desirable to complete the treatment in a short period of time. To achieve this, trials comparing the efficacy and side effects of different fractions, such as 1, 5, or 10 fractions, are warranted.

## Availability of data and materials

This article is not a *meta*-analysis. Therefore, each data is described in the reference paper.

## Author’s information

Tanaka is one of the Japanese Society for Radiation Oncology’s representatives and a member of the Palliative Radiotherapy Research Group and Japanese Radiation Oncology Study Group’s Urological Tumor Research Group. Osamu Tanaka: https://researchmap.jp/osa0508?lang = en.

## Declaration of competing interest

The authors declare that they have no known competing financial interests or personal relationships that could have appeared to influence the work reported in this paper.

## References

[1] Thrumurthy S.G., Chaudry M.A., Chau I., Allum W. (2015). Does surgery have a role in managing incurable gastric cancer?. Nat Rev Clin Oncol.

[2] Pereira J., Phan T. (2004). Management of bleeding in patients with advanced cancer. Oncologist.

[3] Kim Y.I., Choi I.J. (2015). Endoscopic management of tumor bleeding from inoperable gastric cancer. Clin Endosc.

[4] Kim Y.I., Choi I.J., Cho S.J., Lee J.Y., Kim C.G., Kim M.J. (2013). Outcome of endoscopic therapy for cancer bleeding in patients with unresectable gastric cancer. J Gastroenterol Hepatol.

[5] Koh K.H., Kim K., Kwon D.H., Chung B.S., Sohn J.Y., Ahn D.S. (2013). The successful endoscopic hemostasis factors in bleeding from advanced gastric cancer. Gastric Cancer.

[6] Katano A., Yamashita H. (2021). The efficacy of hemostatic radiotherapy for advanced malignancies assessed by World Health Organization bleeding status. Cureus.

[7] Tey J., Back M.F., Shakespeare T.P., Mukherjee R.K., Lu J.J., Lee K.M. (2007). The role of palliative radiation therapy in symptomatic locally advanced gastric cancer. Int J Radiat Oncol Biol Phys.

[8] Lee Y.H., Lee J.W., Jang H.S. (2017). Palliative external beam radiotherapy for the treatment of tumor bleeding in inoperable advanced gastric cancer. B.M.C. Cancer.

[9] Tey J., Choo B.A., Leong C.N., Loy E.Y., Wong L.C., Lim K. (2014). Clinical outcome of palliative radiotherapy for locally advanced symptomatic gastric cancer in the modern era. Med (Baltim).

[10] Kawabata H., Uno K., Yasuda K., Yamashita M. (2017). Experience of low-dose, short-course palliative radiotherapy for bleeding from unresectable gastric cancer. J Palliat Med.

[11] Tey J., Soon Y.Y., Koh W.Y., Leong C.N., Choo B.A., Ho F. (2017). Palliative radiotherapy for gastric cancer: a systematic review and meta-analysis. Oncotarget.

[12] Lee J.A., Lim D.H., Park W., Ahn Y.C., Huh S.J. (2009). Radiation therapy for gastric cancer bleeding. Tumori.

[13] Chaw C.L., Niblock P.G., Chaw C.S., Adamson D.J. (2014). The role of palliative radiotherapy for haemostasis in unresectable gastric cancer: a single-institution experience. Ecancermedicalscience.

[14] Hashimoto K., Mayahara H., Takashima A., Nakajima T.E., Kato K., Hamaguchi T. (2009). Palliative radiation therapy for hemorrhage of unresectable gastric cancer: a single institute experience. J Cancer Res Clin Oncol.

[15] Asakura H., Hashimoto T., Harada H., Mizumoto M., Furutani K., Hasuike N. (2011). Palliative radiotherapy for bleeding from advanced gastric cancer: is a schedule of 30 Gy in 10 fractions adequate?. J Cancer Res Clin Oncol.

[16] Kondoh C., Shitara K., Nomura M., Takahari D., Ura T., Tachibana H. (2015). Efficacy of palliative radiotherapy for gastric bleeding in patients with unresectable advanced gastric cancer: a retrospective cohort study. BMC Palliat Care.

[17] Kosugi T., Shikama N., Saito T., Nakamura N., Nakura A., Harada H. (2016). A nationwide survey in Japan of palliative radiotherapy for bleeding in gastrointestinal and genitourinary tumor patients. World J Oncol.

[18] Corry J., Peters L.J., D'Costa I., Milner A.D., Fawns H., Rischin D. (2005). The ‘QUAD SHOT’–a phase II study of palliative radiotherapy for incurable head and neck cancer. Radiother Oncol.

[19] Tey J., Zheng H., Soon Y.Y., Leong C.N., Koh W.Y., Lim K. (2019). Palliative radiotherapy in symptomatic locally advanced gastric cancer: a phase II trial. Cancer Med.

[20] Saito T., Kosugi T., Nakamura N., Wada H., Tonari A., Ogawa H. (2022). Treatment response after palliative radiotherapy for bleeding gastric cancer: a multicenter prospective observational study (JROSG 17–3). Gastric Cancer.

[21] Tanaka O., Sugiyama A., Omatsu T., Tawada M., Makita C., Matsuo M. (2020). Hemostatic radiotherapy for inoperable gastric cancer: a pilot study. Br J Radiol.

[22] Urquhart R., Scruton S., Kendell C. (2022). Understanding cancer survivors’ needs and experiences returning to work post-treatment: a longitudinal qualitative study. Curr Oncol.

[23] Song I.J., Kim H.J., Lee J.A., Park J.C., Shin S.K., Lee S.K. (2017). Clinical outcomes of endoscopic hemostasis for bleeding in patients with unresectable advanced gastric cancer. J Gastric Cancer.

[24] Meeuse J.J., van der Linden Y.M., van Tienhoven G., Gans R.O., Leer J.W., Reyners A.K. (2010). Efficacy of radiotherapy for painful bone metastases during the last 12 weeks of life: results from the Dutch Bone Metastasis Study. Cancer.

[25] Kawabata H., Fujii T., Yamamoto T., Satake H., Yamaguchi K., Okazaki Y. (2022). Palliative radiotherapy for bleeding from unresectable gastric cancer using three-dimensional conformal technique. Biomedicines.

[26] Lordick F., Carneiro F., Cascinu S., Fleitas T., Haustermans K., Piessen G. (2016). Gastric cancer: ESMO clinical practice guideline for diagnosis, treatment and follow-up. Ann Oncol.

[27] Kim M.M., Rana V., Janjan N.A., Das P., Phan A.T., Delclos M.E. (2008). Clinical benefit of palliative radiation therapy in advanced gastric cancer. Acta Oncol.

[28] Hiramoto S., Kikuchi A., Tetsuso H., Yoshioka A., Kohigashi Y., Maeda I. (2018 Dec). Efficacy of palliative radiotherapy and chemo-radiotherapy for unresectable gastric cancer demonstrating bleeding and obstruction. Int J Clin Oncol.

[29] Lee J., Byun H.K., Koom W.S., Lee Y.C., Seong J. (2021 Aug 23). Efficacy of radiotherapy for gastric bleeding associated with advanced gastric cancer. Radiat Oncol.

[30] Yu J., Jung J., Park S.R., Ryu M.H., Park J.H., Kim J.H. (2021 Apr 15). Role of palliative radiotherapy in bleeding control in patients with unresectable advanced gastric cancer. BMC Cancer.

[31] Mitsuhashi N., Ikeda H., Nemoto Y., Kuronuma M., Kamiga M., Hiroshima Y. (2021 Dec 22). Hemostatic effect of palliative radiation therapy in preventing blood transfusions from bleeding occurring within advanced gastric cancer. Palliat Med Rep.

[32] Takeda K., Sakayauchi T., Kubozono M., Katagiri Y., Umezawa R., Yamamoto T. (2022 Apr 12). Palliative radiotherapy for gastric cancer bleeding: a multi-institutional retrospective study. BMC Palliat Care.

[33] Sugita H., Sakuramoto S., Mihara Y., Matsui K., Nishibeppu K., Ebara G. (2022 Jun). Verification of the utility of palliative radiotherapy for hemostasis of gastric cancer bleeding: a case control study. J Gastrointest Cancer.

